# The experiences of Chinese general practitioners in communicating with people with type 2 diabetes—a focus group study

**DOI:** 10.1186/s12875-021-01506-9

**Published:** 2021-07-19

**Authors:** Mi Yao, Dong-ying Zhang, Jie-ting Fan, Kai Lin, Shamil Haroon, Dawn Jackson, Hai Li, Wei Chen, Richard Lehman, Kar Keung Cheng

**Affiliations:** 1 Institute of Applied Health Research, University of Birmingham, Birmingham, B15 2TT UK; 2 Guangzhou Institute of Respiratory Health, The First Affiliated Hospital of Guangzhou Medical University, Guangzhou, China; 3 Department of Endocrinology, PLA Rocket Force Characteristic Medical Center, Beijing, China; 4 Family Medicine Center, The First Affiliated Hospital of Shantou University Medical College, Shantou, China; 5 College of Medical and Dental Sciences, University of Birmingham, Birmingham, UK; 6 Department of Endocrinology, The First Affiliated Hospital of Sun Yat-Sen University, Guangzhou, China; 7 Department of Nephrology, The First Affiliated Hospital of Sun Yat-Sen University, Guangzhou, 510 080 China

**Keywords:** Type 2 diabetes mellitus, Communication, Experiences, General practitioner

## Abstract

**Background:**

China has more ascertained cases of diabetes than any other country. Much of the care of people with type 2 diabetes (T2DM) in China is managed by GPs and this will increase with the implementation of health care reforms aimed at strengthening China’s primary health care system. Diabetes care requires effective communication between physicians and patients, yet little is known about this area in China. We aimed to explore the experiences of Chinese GPs in communicating with diabetes patients and how this may relate to communication skills training.

**Methods:**

Focus groups with Chinese GPs were undertaken. Purposive sampling was used to recruit 15 GPs from Guangzhou city in China. All data were audio-recorded and transcribed. A thematic analysis using the Framework Method was applied to code the data and identify themes.

**Results:**

Seven males and 8 females from 12 general practices attended 4 focus groups with a mean age of 37.6 years and 7.5 years’ work experience. Four major themes were identified: diversity in diabetic patients, communication with patients, patient-doctor relationship, and communication skills training. GPs reported facing a wide variety of diabetes patients in their daily practice. They believed insufficient knowledge and misunderstanding of diabetes was common among patients. They highlighted several challenges in communicating with diabetes patients, such as insufficient consultation time, poor communication regarding blood glucose monitoring and misunderstanding the risk of complications. They used terms such as “blind spot” or “not on the same channel” to describe gaps in their patients’ understanding of diabetes and its management, and cited this as a cause of ineffective patient-doctor communication. Mutual understanding of diabetes was perceived to be an important factor towards building positive patient-doctor relationships. Although GPs believed communication skills training was necessary, they reported rarely received this.

**Conclusions:**

Chinese GPs reported facing challenges in communicating with diabetes patients. Some of these were perceived as being due to the patients themselves, others were attributed to system constraints, and some were seen as related to a lack of clinician training. The study identified key issues for the development of primary care-based management of diabetes in China, and for developing appropriate communication skills training programs for the primary care workforce.

**Supplementary Information:**

The online version contains supplementary material available at 10.1186/s12875-021-01506-9.

## Background

The detected prevalence of type 2 diabetes mellitus in China has grown rapidly, from 1% in the 1980s to 10.9% in 2013. It is now estimated that 114 million Chinese people have the condition [1]. The cost of diabetes management in China is predicted to exceed RMB 360 billion (almost USD 51 billion) annually by 2030 [2]. It is imposing a huge economic burden for both patients and the wider society in China. Furthermore, the diagnosis, treatment, and control of diabetes are currently not optimal, and very few patients are prescribed drugs to prevent cardiovascular disease, particularly antihypertensive drugs and statins [1, 3]. In addition, the burden of multimorbidity in patients with diabetes is rising with the increasingly ageing population [4]. Multimorbidity has brought on additional challenges for diabetes self-management and has increased pressures on the healthcare system. Against this background, there is an increasing awareness that the current care model for diabetes is unsustainable, with over-reliance on hospital care and relatively weak performance in primary care.

In response to such challenges, the Chinese government has committed to a dramatic increase in the capacity of the primary health care system [5]. This includes designing integrated care pathways between primary and secondary care, alongside training for general practitioners (GPs) [6, 7]. The aim is to train up to 400,000 new GPs by 2030, to produce a total workforce of 700,000, equivalent to 2–3 per 1,000 population [8]. There are two main training pathways in China: a 5-year undergraduate program with 3 years of residency training (“GP residency training”) and a transfer training pathway for community hospital-based physicians to become GPs within 1–2 years (“GP transfer training”) [9]. Additionally, universal health insurance coverage, a basic public health service program, and a national essential drug system, were developed by the government to improve access and affordability in primary health care. The primary healthcare system was seen as a means of addressing the burden of chronic non-communicable diseases in the government’s Healthy China 2030 plan [10]. As a result, much of the care of patients with T2DM is likely to move into primary care.

Despite increased financial investment and favorable policies in strengthening primary care in China, poor quality of care for chronic non-communicable diseases (such as diabetes) still exists, with fragmentation insufficient continuity of clinical care. Primary care in China usually does not provide the first point of care and infrequently coordinates with specialty care. Both hospitals and general practices are paid by a fee-for-service related to the care they complete for patients [10]. Within the social health insurance program (a 70% government subsidy and 30% individual premium) patients are reimbursed wherever they seek care without referral [11]. This is in contrast to other healthcare systems in which primary care acts as the gatekeepers to secondary care services.

Diabetes care requires effective communication between physicians and patients. This can enhance their cooperation, and is associated with increased understanding of treatment, adherence to recommendations, patient satisfaction, and improved clinical outcomes [12, 13]. Diabetes patients who are more engaged with their doctors and more involved in decision making are shown to comply better with medical recommendations and self-care activities [14]. However, in China, recent studies have found that poor communication and relationships between doctors and patients has led to a low level of trust [15–17]. Communication skills training could help to improve this. However, communication skills training is currently rarely provided in medical schools, or in continuing medical education for residents and practicing physicians in China [18].

To our knowledge, no studies have explored the experiences of Chinese GPs in communicating with diabetes patients. We therefore undertook a qualitative study to address the following: (a) To explore the perceptions of GPs, particularly in relation to their experiences of communicating with diabetes patients, doctor-patient relationships, and the socio-cultural context impacting on diabetes care and self-management; (b) To explore GPs perceptions on communication skills training in this area. We identify elements of communication which might be improved by a training program, and also look at doctors' experience of trying to communicate with their diabetes patients from a wider systems perspective. This consideration of the socio-cultural context will help explore the contribution of the current health systems and state of primary care in China to diabetes care, and to the experience of doctors delivering that care.

## Method

### Study design and participants

We conducted a qualitative study using focus groups. Focus groups were chosen to reduce the impact of any social distance between the facilitator and participants on the discussions, and to explore complex problems and shared experiences with group interaction [19, 20]. GPs working in general practices in Guangzhou, China were eligible to participate. Guangzhou is a modern industrial city located in the South of China. It is the capital city of Guangdong province with close to fifteen million urban residents at the end of 2019 [21]. There were 188 community healthcare service centers (general practices) with about 5000 GPs, 303 secondary hospitals and 70 tertiary hospitals in 2021 [22]. The study was advertised (through paper and electronic invitations) at various GP seminars and conferences in Guangzhou, outlining the research background, aims and methods. GPs were invited to express interest in the study by contacting the focus group facilitator (MY) by email or WeChat, and providing basic demographic information. Participants were purposively sampled based on their working area (rural or urban), age and years of experience working in primary care [23].

Recruitment was balanced between having an adequate number of participants to be able to draw conclusions and stopping recruitment when data saturation had been reached. Data saturation was defined as no new codes and no new significant themes being identified from subsequent data. A flexible topic guide was used to stimulate open discussion, while ensuring key issues were covered in investigating the experience of GPs in communicating with patients with diabetes. The focus group guide was developed using the findings of a systematic review together with discussions with the multidisciplinary and multi-national team involved in this research [24]. (see Table 1).

**Table 1:** Focus group discussion guide for GPs

1. Issues of confidentiality and anonymity
Reinforce written participant information, emphasizing that no participant would be identifiable in any dissemination or publication of the study by the investigators. Establish ground rules for participants. Advise participants to draw the group’s attention to any information that they do not wish to be repeated outside the group by other participants in any further discussions. Confirm consent to audio-recording
2. Prompts for facilitators
What do you think are the most important things for diabetes patients? (HbA1c, blood pressure, quality of life, etc.)
What is your experience in communicating with diabetes patients?
Are there any barriers (gaps) or facilitators in communication with diabetes patients?
How do you feel when your diabetes patients present with emotional difficulties?
How do you see your attitudes and behaviors (words, emotion and expression styles) affecting your diabetes patients' self-care?
What do you think make diabetes patients trust doctors?
Are there any good communication skills in daily practice with diabetes patients?
Have you received any communication skills training before? If yes, what is your experience in communication skills training, e.g., training content and methods?
Do you think training will help improve GPs communication skills? If yes, why?
Is there anything else about the physician/patient relationship that you want to share?

The recruitment process and focus groups took place from November 2019 to April 2020. All GP participants provided written informed consent and completed a questionnaire to collect demographic information including age, gender, years in practice, education background and location of practice. One researcher (MY) conducted all the focus groups as a facilitator, and another researcher (DZ) acted as a co-facilitator. Both researchers were trained in qualitative research methods and had no prior relationship with any of the participants. The facilitators reflected that participants were engaged, generous and authentic. Field notes were made by one researcher after each focus group (MY or DZ). All focus groups were held at the First Affiliated Hospital of Sun Yat-sen University, a central point in Guangzhou that is easily accessible by public transport as well as by car. A compensation of a RMB 200 (equivalent to 28 US dollars) shopping voucher was offered to participants to reimburse travel costs. Ethical approval was provided by the Medical Ethics Committee of The First Affiliated Hospital of Sun Yat-sen University (Reference number [2019]369).

### Analysis

Audio-recorded data were transcribed verbatim and reviewed for accuracy by two researchers (MY & JF). One focus group transcript was randomly selected by researchers and returned to participants for comments to validate the transcription process. No correction were required for this transcript. Anonymized transcripts were imported into NVivo12 software and coded independently by two researchers (MY & DZ). Thematic analysis was undertaken using the Framework Method [25]. Analysis was ongoing and iterative, informing further data collection. For the first stage of the thematic analysis, two researchers independently read two random focus group transcripts and field notes and open-coded the data. Key words and phrases were used as the units of analysis to generate initial codes. Meaning units from the transcripts were discussed and condensed to a description close to the context. Discrepancies and disagreements were resolved through discussion and consensus to develop the initial thematic framework, which was then applied to all remaining transcripts. Once all data had been coded using this framework, we summarized the data in a matrix based on similarities and differences of codes. Sub-themes were generated from the data set by reviewing the matrix and making connections within codes. Themes and sub-themes were identified until data saturation had been reached. The analysis and interpretation of the data were discussed by authors and disagreements resolved by consensus. Relevant quotations were identified and selected from the transcripts to highlight the themes. Findings were provided to four participants in one focus group for review, and they all agreed that this accurately reflected their discussions. This study was reported according to the 32-item checklist of Consolidated Criteria for Reporting Qualitative Research (COREQ) [26]. (see Additional file).

The first author (MY, male) is a practicing general practitioner in China and undertaking a PhD in medicine in the UK. DZ (female) is an academic researcher with relevant expertise in primary health care in China.

## Results

Four focus group discussions with 15 GPs from 12 general practices in Guangzhou (mean duration 58 min, range 50 to 86) were held and no participants dropped out. See Table 2 for GP characteristics and focus group information.

**Table 2:** Focus group characteristics: gender, age, education background, previous GP training experience, and location of practices in Guangzhou^a^ (n = 15)

	Focus group 1 (N = 4; M1, F3)	Focus group 2 (N = 3; M2, F1)	Focus group 3 (N = 4; M1, F3)	Focus group 4 (N = 4; M3, F1)
Participant 1	29, 3, E1, T1, D1	35, 6, E1, T1, D2	43, 10, E1, T2, D1	39, 8, E1, T2, D1
Participant 2	31, 4, E2, T1, D1	40, 10, E1, T2, D2	50, 12, E3, T2, D1	41, 10, E1, T2, D1
Participant 3	37, 8, E1, T2, D1	36, 7, E1, T1, D2	42, 9, E1, T2, D1	32, 5, E2, T1, D1
Participant 4	30, 4, E1, T1, D1		33, 5, E1, T1, D2	46, 11, E1, T2, D1

^a^Gender (M/F), Age (years), GP experience (years worked as GPs), Education background (E1-E3, E1 Bachelor’s degree, E2 Master’s degree, E3 College degree), Previous GP training experience (T1-T2, T1 GP residency training, T2 GP transfer training), District in Guangzhou (D1-D2, D1 city center, D2 rural or suburb)

Four main themes were identified from the focus groups: diversity in diabetic patients, communication with patients, patient-doctor relationship and communication skills training. The themes and subthemes are presented in Table 3.

**Table 3:** Themes and subthemes

Themes	Subthemes
1. Diversity in diabetic patients	a. Diabetes patients’ attitudes, knowledge, and behavior
	b. Medication adherence
	c. Patients’ emotional problems
2. Communication with patients	a. Consultation management
	b. Blood glucose monitoring and control
	c. Communication difficulties and facilitators
3. Patient-doctor relationship	a. Mutual understanding
	b. Blaming doctors
	c. Blurring of the boundaries
4. Communication skills training	a. Insufficient training
	b. Training needs
	c. Practice and feedback

### Theme 1: Diversity in diabetic patients

#### Diabetes patients’ attitudes, knowledge, and behavior

Patients with diabetes were described by GPs as often being in denial of their diagnosis, expressing fear or anxiety, losing patience and even giving up. A number of factors were perceived to affect how patients view their condition. The GPs described that some asymptomatic patients did not take their diagnosis of diabetes seriously while those with obvious or severe symptoms (e.g., itchy skin), or complications (e.g., diabetic retinopathy), were often worried and concerned. Patients with longstanding diabetes worried about their bodily function such as their liver and renal function. Some patients worried about the dietary and life-style changes required to self-manage their condition, although participants acknowledged that some young patients were more willing to engage in dietary and lifestyle changes, rather than taking medication. Some patients also worried about diabetes being inherited in their families. However, some well-controlled patients with long term diabetes were described by GPs as having an optimistic attitude and confidence in living with diabetes.

‘The patient cannot accept that he has diabetes, and he cannot accept it psychologically, and he denied that he had the disease.’ (FG [focus group]2 P2).

‘Some patients had concerns about complications that might affect them, for example, some patients had diabetic feet, and then they worried about whether they might have to have an amputation or other problems because of the infection. In some cases, because of the long-term effects of diabetes on vision, there was a serious concern about becoming blind.’ (FG1 P1).

‘Not all of them are worried about their diabetes. Some well-controlled patients often told me about their diabetes experiences, such as regular exercise and a healthy diet. I think they are very optimistic.’ (FG1 P2).

GPs described that the majority of patients’ knowledge about diabetes was insufficient and that misunderstanding was common. However, most patients wanted to know how diabetes might progress and the associated risks, especially those with other long term conditions. Participants reported several factors that may affect patients’ understanding of diabetes and their health literacy, such as being in contact with other diabetes patients, family members, access to health information, and socioeconomic factors. Some physicians felt that doctors themselves carried some responsibility for patients’ poor knowledge of diabetes as a result of ineffective communication with patients. However, some participants mentioned that patients with longstanding diabetes had a considerable amount of diabetes related knowledge, that sometimes exceeded that of young doctors.

'Patients are very short of knowledge about diabetes, such as how to monitor blood glucose, how to take drugs, whether to take drugs before or after a meal, the harm of diabetes, and matters needing attention in exercise and diet control. All of which are lacking.' (FG1 P2).

‘Many patients who come to see me really want to know the prognosis of the disease, how serious the disease is, and what is the risk for the implications.’ (FG1 P1).

‘Some patients thought that the doctor's words are not as useful as the neighbor's words. What medicine the neighbor told him to take, he immediately went to the pharmacy to buy it. The neighbor said that a certain medicine can lower blood sugar, he bought it immediately.’ (FG1 P4).

‘Some patients, especially in the ‘villages’ in the city, they are very young and unable to read and write, even those in their 30 s or 40 s who were not able to write their own names. In the face of such a patient, I think it is impossible to simply expect him to understand the complications of diabetes.’ (FG3 P3).

‘Patients who have been treated at hospitals or community centers for more than five years are well aware of the symptoms, harms, and complications of diabetes. They know more about diabetes than younger doctors.’ (FG4 P3).

GPs described some patients as “lazy” and unwilling to make lifestyle change even when knowing the risk of diabetes, and that this applied especially to young patients. However, some patients looked up information for themselves and compared different information sources through the internet. Some physicians also described two kind of diabetes patients: “pseudo experts” and the “deceived person”. The “pseudo expert” patients consulted the internet, placed significant authority on what they discovered, and perceived themselves to be sufficiently informed on the management of their condition. They frequently asked their doctors to make prescriptions for treatment during the consultation (“like ordering food at a restaurant or supermarket”). Patients described as the “deceived person” were perceived to be unable to independently analyze and assess incorrect health information and were sometimes tricked into buying health supplements that had no therapeutic benefit.

‘Even if they face the risk of diabetes, sometimes they are really reluctant to make some lifestyle changes.’ (FG2 P1).

‘He (patient) found some health products information from the WeChat Moments (online social platform) or found some home remedies and diets in other places, and then wrote them on paper. And he brought this paper to me and asked me to follow his mixed treatment plan on diabetes. But in fact, when I told him something more authoritative, he did not understand’ (FG4 P4).

#### Medication adherence

GPs recognized that most patients were taking multiple medications. Medication frequency, duration and price were thought to greatly affect patients’ medication adherence. Patients were perceived to be concerned about both the effectiveness and the side-effects (e.g., liver and kidney impairment, hypoglycemia, etc.) of diabetes medication, especially among older patients. Many GPs had found that patients refused to take insulin due to a fear of needles and a feeling that using insulin means they had “failed” at managing their diabetes. They gave accounts of patients who were being treated with insulin, yet had asked their doctors to switch them to oral medication or to simply discontinue insulin. Some young patients refused to take medication and preferred exercise and dietary changes to control their condition. GPs also recounted complaints from patients who wished their prescriptions could be issued for longer than monthly as a lot of time was spent travelling to practices and waiting for consultations.

‘Especially if you want to persuade patients to take insulin, they are even more afraid. They feel that once they use insulin, they cannot stop it and have to use it all the time’ (FG1 P2).

‘For example, the drug Sitagliptin, because it can be taken one tablet a day, many patients like to use it. But for Acarbose, which is taken three times a day, seems to be too much trouble, and it is not acceptable. Patients like the simple way of taking medicine.’ (FG3 P1).

#### Patients’ emotional problems

GPs described that some patients had emotional problems, or problems such as anxiety, depression, or other mental health disoders. Most of these problems were considered associated with economic and family issues which,in turn, affected patients’ attitudes and behaviors to self-manage their diabetes. GPs felt that some patients saw doctors mainly as a source of comfort for their emotional problems. Although GPs recognized that some emotional issues could be resolved by finding solutions together with patients, they found it was very difficult to manage their mental health. In turn, it was also recognized that patients’ mood could also affect doctors. Most physicians mentioned that there were no tools to evaluate diabetes patients’ psychological or mental health problems in clinical encounters. However, some physicians mentioned that they would refer patients to psychologists or diabetes specialists, and this could relieve patients’ emotional problems to some extent during the consultation.

‘Of course, if the patient is uncomfortable, I can feel it directly. Many diabetes patients cried in my consultation room.’ (FG4 P3).

‘Because we do not have our own diagnosis and treatment system, and do not have the matching evaluation tools, I can only say that I can evaluate the emotional state of diabetes patients based on my own feelings.’ (FG1 P2).

### Theme 2: Communication with patients

#### Consultation management

GPs described that their consultations with diabetes patients were not by prior appointment, which often caused patients to wait for a long time and doctors to be hurried when communicating with them. Normally, consultation times are very short, ranging from three to five minutes. Patients were perceived to be afraid to ask their doctors too many questions as they knew doctors had no time to answer them. However, some GPs mentioned that providing patient information leaflets on diabetes was helpful and could improve time management during consultations.

Online communication (e.g., Wechat, a mobile phone application) was used by most of physicians to answer questions without the need for direct face-to-face consultations. Physicians typically built an online WeChat group of about 100 to 500 patients. When patients had any questions, they could ask questions in these online forums. Other patients in these groups were thought to benefit from these online conversations, providing an opportunity for them to find useful information. However, some physicians did not agree with this method and believed that face-to-face communication was better than online, especially in long term management and follow-up.

The most difficult thing for GPs was to acquire patients’ health records from other hospitals or clinics. Patients often could not remember their own health information and (for those in possession of a health record) did not bring it with them.

‘Frankly speaking, sometimes I'm really scared that I don't have enough time. I personally feel that if I have time to talk to patients with diabetes under current circumstances, I can do my best. But in fact, there is no more time for me, and it is really difficult to do more for patients. It really takes extra time to comfort the patient.’ (FG3 P4).

‘I designed a blood glucose book by myself and made a grid for patients. I provided this piece of paper to them. I told them which monitoring points and saying that I hope you(patients) can do next time. I gave them this form to make it like homework. If the patient does what I want, I think this paper can serve as a supervision. I think this is a method for patients self-management and for me to know their control.’ (FG2 P2).

#### Blood glucose monitoring and control

Most GPs described that blood glucose monitoring and control was very important and they often set goals for patients. Guidelines and clinical pathways require doctors to monitor patients’ glucose as an indicator to evaluate the quality of diabetes care and to screen for diabetes. However, the GPs’ felt that many patients were unwilling to have blood glucose tests because they found tests painful. By contrast, other patients checked their blood glucose frequently as they were worried about their glucose variability. Participants reported spending a lot of time explaining glucose control.

‘Many patients are used to checking their fingertip blood glucose several times a month. Frankly speaking, the figures changed all the time. Patients are very nervous. They will say why it is high, whether it is the problem of taking drugs, and then this caused the patients to have some bad emotions, and then doctors have to deal with. Fluctuations in blood glucose do cause some unnecessary troubles and increase the amount of time we need to explain to patients each time.’ (FG4 P1).

#### Communication difficulties and facilitators

GPs described several difficulties in communicating with diabetes patients, including lifestyle change, dietary change, discussing risk of complications, medication change, referring to specialists and giving bad news. Some physicians used the expressions “blind spots” or “not being on the same channel” with their patients. These terms referred to situations where doctors and patients had conflicts of understanding, and these sometimes caused disputes between doctors and patients. A common phenomenon was that patients often had different treatment plans (some with traditional Chinese medicine) from the different doctors they visited, especially diabetes specialists, which made it difficult for GPs to decide which plan should be followed when communicating with patients. Patients often placed more trust in treatment plans from specialists than from GPs. Some GPs mentioned that patients were unwilling to talk with young or new doctors.

‘We often have some blind spots in communication with patients. Sometimes we may be clearly for the sake of their good, but we may not speak and express well, so that they do not understand, and may even cause us to dispute’ (FG3 P1).

‘Sometimes words from specialists in hospitals were more useful than we said. If specialists give some treatment plans, the patient may say that the plan should be implemented all the time. When we communicate with the patient afterwards, patients always listen to the specialists and feel that our plan is wrong.’ (FG1 P3).

‘When sharing bad news, such as telling the patient when he will die, or amputation, or his vision will be permanently blind, or his energy will not recover in the future. In these cases, it is difficult to tell him and let him accept such bad information.’ (FG4 P2).

The GPs did describe some methods to promote communication in clinical encounters. Respecting patients’ choices, agreeing and encouraging patients, providing patients’ opportunities to express and ask questions, aiming to understand what patients were thinking, learning patients’ characters, using examples, and making decisions together, were described as facilitating communication. Empathy, maintaining eye contact, listening, using a polite tone and plain language were described as effective communication skills. Some physicians expressed that offering patients small gifts or free services, such as free blood glucose tests or insulin needles (as patients usually have to pay for these), were helpful in promoting communication. Some physicians thought that panicking patients was useful, such as showing patients pictures of diabetes foot ulceration, while some physicians believed that this way would unduly worry patients.

‘Sometimes I will praise them (patients) in front of their families, they will feel a sense of honor and pride. In short, in some situations like this, with timely encouragement and prompt praise, they will more easily accept my suggestions’ (FG2 P3).

‘Sometimes when I try to get to know my patients, to allow them to express their feelings, to respect their choices and to make decisions together, it makes our communication process more harmonious. I think that's how you get both sides on the same channel.’ (FG2 P4).

‘Tell them (patient) what is the danger of diabetes, but maybe because my way of expressing is not very good, they don’t take it seriously. On the contrary, showing them some horrible pictures or video materials will impress them. I think this is an important communication skill.’ (FG3 P2).

Some GPs expressed the feelings they experienced when communicating with diabetes patients. When they saw the condition of their patients was poorly managed, they felt sad or experienced a sense of failure. In contrast, if patients’ diabetes was controlled well, they felt happy and had a sense of accomplishment. They also felt a sense of loss and lack of respect when patients compared them negatively with diabetes specialists.

‘Our GPs have a sense of frustration and failure. If he (patient) went to tertiary hospitals, he might be very obedient. Subconsciously, he may feel that the doctors in the tertiary hospitals are better than the doctors in our general practice.’ (FG4 P2).

### Theme 3: Patient-doctor relationship

Some GPs described that first impressions and mutual understanding were important factors to build patient-doctor relationships. They also expressed that several factors could affect patients’ trust in their doctor. The better their professional qualifications, professionalism, self-confidence and communication skills, the more they felt patients tended to trust them. However, GPs also reported negative patient-doctor relationships. They felt that patients may complain or blame doctors if the consultation time was short or their conditions were not managed well. GPs were very unclear whether the responsibility for the latter fell on the clinician or the patient. Some GPs mentioned that patients believed doctors were making money from them from prescriptions and by offering tests, and even by deliberately over-prescribing and over-testing. However, some GPs mentioned that they had a good relationship with patients, as multiple consultations built trust. They even worried whether a close patient-doctor relationship could potentially be harmful, as it could cause blurring of the boundaries of the patient-doctor relationship, seeing patients almost as their relatives and stepping too far into their patients’ lives.

‘Trust is built in two ways, one is effective communication, and another is effective treatment. If you said well, but his blood glucose does not fall, he will not believe you. Therefore, I think we should convince him with professional knowledge, from the aspects of weight management of his diet to medication. And if he can cooperate with my suggestions, I think it is possible to achieve mutual trust.’ (FG1 P1).

‘In fact, I think that if one patient follows you for a long time, sometimes it will give you an illusion that he is already your loved one or family member. Then when you are on holiday or some time you will think that he might eat too much, and his blood glucose is not good. It is really an illusion to have a long relationship with people with diabetes. It’s hard to say whether this feeling is good or not.’ (FG3 P4).

### Theme 4: Communication skills training

Almost all the GPs stated that they had seldom received any communication skills training in medical school, or later in their continuing medical education. They acknowledged that communication skills were not a natural ability and needed training. They hoped communication skills training programs for them would be framed in the everyday reality of clinical practice rather than on theories alone. Being able to participate and receive feedback was perceived to be helpful. Some physicians suggested role-play as a form of training, while other physicians did not agree as they could not transfer role-play into real practice since they were not “actors in a TV show.”

‘Basically, there is very little relevant training in this area. There are many details about how to establish some such relationship, communication skills with the patients, how to gain the trust of patients, how to communicate with the patient, such trainings for us are rare.’ (FG3 P3).

‘That's something I need to learn. It's not like I can do it by taking a few classes or lectures. I may understand everything in class, but I am not able to do it in practice. It needs to be practiced repeatedly to achieve the best.’ (FG1 P3).

## Discussion

To better understand the experiences of Chinese GPs in communication with diabetes patients, we undertook a focus group study. Our questions encouraged the participants to talk openly about the issues that they felt affected communication with this patient group. They responded by commenting about the context as well as the content of consultations for diabetes in the current state of primary care in China. We did not wish to limit their input to aspects which might be remediable by communication skills training alone. Instead we sought to explore all the barriers and facilitators which these doctors encountered as part of their whole experience of delivering care. A major theme was uncertainty about their role and status, and the impossibility of achieving adequate communication in the consultation time available. These are systems challenges for the whole of Chinese primary care at present. Another major theme was the great diversity of patient understanding and responsiveness. It is clear that primary care doctors cannot address this by themselves, and that this is therefore also a systems challenge for better patient education, self-management and team care.

It is surprising that some physicians in focus groups called their diabetes patients “pseudo experts”. This term was not found in previous literature. The physicians in the study believed that patients had too much faith in their own knowledge while the authority of physicians was not respected. These patients may want to have more autonomy, but the participants found this difficult to cope with. Once an antagonistic approach was established, it would be a great barrier to doctor-patient communication and relationship. An alternative view was that patients seeking information from the internet should not be labelled “pseudo-experts” but as collaborators with their GPs in finding evidence-based sources of diabetes information to help them manage their condition. We also found that most GPs in focus group described “blind spots” or “not being on the same channel” with their patients. In fact, patients and doctors are two kind of experts [27]. Patient experts know themselves well and have their own attitudes, values, and preferences in diabetes care. Doctor experts know the evidence base and can advise on the potential pros and cons of different treatment regimens [28]. Current diabetes care in primary care in China might benefit from a change of attitude towards patients, away from a paternalistic model of obedience to standard advice, towards a model of partnership towards finding individual solutions.

Almost all the GPs described that their patients saw blood glucose control as very important, and management of this was often set as the goal of diabetes care for patients and their GP. However, our study showed that using numerical targets made both patients and doctors worried in communication. Patients worried about their condition fluctuating or worsening, and doctors worried about how to explain the figure changing. The problem arises because figures can be easily measured in a very short clinical encounter and too much reliance is placed on them in clinical guidelines and pathways in the Chinese primary care health system. It would be helpful if there were some specific patient tools to promote better discussions between patients and doctors. Doctors also need better guidance and permission to move away from this predominantly glucocentric view. There is increasing global consensus that diabetes care should be centred on the individual needs of patients according to their personal risk profile and their informed preference for management options [29].

In our focus group study, most GPs rarely received communication skills training. This finding was consistent with previous studies [18]. Our report on barriers and facilitators experienced by GPs in communicating with diabetes patients could help to inform future training, especially in a transition from a predominantly secondary to primary care-led service, where large numbers of diabetes patients will receive care. Training could focus on combining traditional communication skills teaching with addressing the practical clinical issues GPs encounter to achieve better patient experience and health outcomes. We also found that referring patients to specialists, negotiating treatment plans between primary and secondary care and patient’s mental health issues are difficult communication areas for GPs. Those issues have been neglected and are not covered in current Chinese diabetes guidelines and clinical pathways [29].

The themes from our study have some similarities with previous studies [30–32]. One systematic review of qualitative studies from developed countries on patients’ and healthcare providers’ perspectives on diabetes management found several themes relating to differences and diverse perceptions between patients and their healthcare providers. Similarly, this showed that providers experience barriers in communication and sometimes lack adequate communications skills, as reported in our study. Both this review and our study found that patients preferred specialists above GPs [24]. These themes present a broader picture of challenges and complexity in communication between GPs and diabetes patients.

To our knowledge, this is the first study to describe the experiences of Chinese GPs in communicating with diabetes patients. A limitation of the study is that the sample was drawn from a single city in China, so it is possible that the views and experiences of physicians from other geographic regions would differ. However, our focus groups encompassed a range of GPs in different general practices, both in urban and rural areas. Another limitation is our focus group numbers are smaller than usually recommended and may have led to idea restriction [33]. However, there are some strengths of smaller groups, including ease of recruitment of GPs, organization and facilitation, and less fragmentation of discussion compared with larger groups. The successful management of diabetes usually depends on more than one clinician and should always involve patients. Future research should therefore explore patients' experience of communicating with GPs.

## Conclusion

Chinese GPs face a wide variety of diabetes patients in their daily practice and encounter many challenges in communicating with them. Some of these are driven by system issues such as short consultation times, lack of patient information resources, inadequate team support, and the perceived low status of primary care in China. While communications skills alone cannot provide a solution to these, better training in dialogue with patients will be needed if primary care is to take on the leading role in caring for China’s 140 million or more patients with diabetes.

## Supplementary Information

**Additional file 1.** COREQ (COnsolidated criteria for REporting Qualitative research) Checklist.

## Data Availability

The anonymized transcribed data from the current study are available from the corresponding author on reasonable request.
